# Modeling transthyretin (TTR) amyloid diseases, from monomer to amyloid fibrils

**DOI:** 10.1371/journal.pone.0304891

**Published:** 2024-06-06

**Authors:** Richard S. Criddle, Lee D. Hansen, Brian F. Woodfield, H. Dennis Tolley

**Affiliations:** 1 Department of Chemistry and Biochemistry, Brigham Young University, Provo, Utah, United States of America; 2 Department of Statistics, Brigham Young University, Provo, Utah, United States of America; Lady Hardinge Medical College, INDIA

## Abstract

ATTR amyloidosis is caused by deposition of large, insoluble aggregates (amyloid fibrils) of cross-β-sheet TTR protein molecules on the intercellular surfaces of tissues. The process of amyloid formation from monomeric TTR protein molecules to amyloid deposits has not been fully characterized and is therefore modeled in this paper. Two models are considered: 1) TTR monomers in the blood spontaneously fold into a β-sheet conformation, aggregate into short proto-fibrils that then circulate in the blood until they find a complementary tissue where the proto-fibrils accumulate to form the large, insoluble amyloid fibrils found in affected tissues. 2) TTR monomers in the native or β-sheet conformation circulate in the blood until they find a tissue binding site and deposit in the tissue or tissues forming amyloid deposits *in situ*. These models only differ on where the selection for β-sheet complementarity occurs, in the blood where wt-wt, wt-v, and v-v interactions determine selectivity, or on the tissue surface where tissue-wt and tissure-v interactions also determine selectivity. Statistical modeling in both cases thus involves selectivity in fibril aggregation and tissue binding. Because binding of protein molecules into fibrils and binding of fibrils to tissues occurs through multiple weak non-covalent bonds, strong complementarity between β-sheet molecules and between fibrils and tissues is required to explain the insolubility and tissue selectivity of ATTR amyloidosis. Observation of differing tissue selectivity and thence disease phenotypes from either pure wildtype TTR protein or a mix of wildtype and variant molecules in amyloid fibrils evidences the requirement for fibril-tissue complementarity. Understanding the process that forms fibrils and binds fibrils to tissues may lead to new possibilities for interrupting the process and preventing or curing ATTR amyloidosis.

## Introduction

ATTR amyloidosis is a protein misfolding disease caused by deposition of large, insoluble aggregates (amyloid fibrils) of cross-β-sheet transthyretin (TTR) protein in a tissue or tissues. Most TTR protein (≈95%, [1, 2]) is synthesized from amino acids in the liver and secreted into the blood stream as a tetramer, (TTR)_4_. TTR amyloidosis from blood borne TTR tetramer develops by a multi-step process of 1) monomer dissociation from the tetramer, 2) monomer folding into a cross-β-sheet, and 3) aggregation into cross-β-sheet fibrils that bind to tissues to form intercellular amyloid deposits [3–6]. The disease phenotype is variable and depends on which tissue is affected. Current treatments for ATTR amyloidosis either decrease synthesis of TTR or stabilize the tetramer in the blood stream, thus lowering the concentration of monomer and slowing disease progression. Understanding the process that forms fibrils and binds fibrils to tissues may lead to new possibilities for interrupting the process and preventing or curing ATTR amyloidosis.

Although much research has been focused on monomer dissociation and the structure of amyloids in tissues, the process from dissociated monomers to tissue amyloids has received little attention. In 2004, Fernandez-Escamilla et al. [7] developed an algorithm (TANGO) to predict the propensity for β-sheet formation from the amino acid sequence and propensity for aggregation of the β-sheets, but concluded, “TANGO predicts β-sheet aggregation and not amyloid formation”. In 2018, Chuang et al. [8] stated, “The mechanisms by which disease proteins aggregate and cause toxicity is not fully understood.” This paper thus focuses on aggregation mechanisms and tissue selectivity based on the concept of molecule-molecule and molecule-tissue complementarity, i.e., the number of attractive interactions minus the number of repulsive interactions. As far as we can determine, no previously published work has considered the implications of a requirement for molecule-tissue or fibril-tissue complementarity.

Two possibilities for the process leading to ATTR amyloidosis are explored here, 1) TTR monomers in the blood spontaneously fold into a β-sheet conformation, aggregate into short proto-fibrils that then circulate in the blood until they find a complementary tissue where they deposit to form the large amyloid fibrils found in the affected tissue or tissues. 2) TTR monomers in the native or β-sheet conformation circulate in the blood until they find a tissue binding site and deposit in the tissue or tissues forming amyloid deposits *in situ*. Note that these models only differ on where the selection for β-sheet complementarity occurs, in the blood or on the tissue surface.

The following summarizes the information relevant to these models that is known about TTR protein, amyloid fibrils, and ATTR amyloidosis.

1. The amino acid sequence in TTR is coded by two equally active genes. Wildtype TTR protein (wt) is produced If the two genes are the same, but if one of the genes is mutated, up to half the TTR protein produced is a variant (v) with one different amino acid. The ratio of wt to v in the blood stream is typically >1 because of differences in secretion efficiency [9].
2. Tissue surfaces are highly organized, unique to a tissue, and unique to an individual [10].
3. TTR protein molecules are bound into amyloid fibrils with multiple weak, non-covalent bonds between the molecules and fibrils are bound to tissues with multiple weak, non-covalent bonds. Total bond strength between TTR molecules and between fibrils and tissues increases as complementarity (i.e., the number of attractive interactions minus the number of repulsive interactions) between the bonding surfaces increases.
4. The table of known variants of TTR and related amyloid diseases [11, 12] shows many examples where both the identity of the amino acid substitution and the position of the substitution affect tissue tropism and thence disease phenotype. Tissues that may be affected by ATTR amyloidosis include the heart, peripheral nervous system, carpal tunnel, central nervous system, kidney, liver, and GI tract [11, 12]. However, wildtype or a given variant TTR that leads to a particular disease phenotype(s) in one individual may cause a completely different disease phenotype or may not cause disease in another individual. When ATTR amyloidosis does occur, amyloid deposition may cause numerous different disorders that have diverse clinical presentations [13]. However, the process that gives rise to these diverse outcomes is not understood [14, 15].

Based on this information, we hypothesize that because tissue surfaces are structured, highly complementary structures of fibrils are also required for stable, insoluble fibrils to form in tissues and cause ATTR amyloidosis. Thus, a statistical model for the probability of tissue selectivity is developed to test the feasibility of this hypothesis.

The surfaces of intercellular spaces where fibrils may bind consist of lipids, proteins, collagen and proteoglycans, etc., that are genetically determined, highly ordered and highly variable among tissues and individuals as shown by graft-versus-host disease. Cells in an organ are bound together by what Shubin [10, pp 127–128] has referred to as “tiny molecular rivets, of which there is a vast diversity”… (these) “molecular rivets are so precise that they bind selectively, only to the same kind of rivet. … (and) organize(s) our bodies in a fundamental way.” These patterns of tissue surface residues offer binding sites for fibrils, but the strength of binding and therefore the probability of ATTR amyloidosis developing depends on the degree of complementarity between the tissue surface and the fibril surface.

To be complementary to a tissue, the fibril surface must also be structured. Fibrils composed of only wildtype TTR cross-β-sheet molecules have a particular surface structure defined by the exposed amino acid side groups and commonly a tendency to bind to heart tissue, but may also bind to peripheral nerve tissues or other tissues [16]. Fibrils composed of both wt and v molecules must have regular repeating structures, i.e., a particular sequence of wt and v, to provide an ordered structure of the exposed amino acid side groups. Selection for complementarity between the cross-β-sheet TTR molecules in mixed fibrils must thus occur to provide the fibril structure for tissue binding.

Fibrils with a particular (fixed) array of amino acid side groups on their surface are here referred to as structured. Fibrils with a fixed sequence of wt and v molecules present mutant-dependent, fixed arrays of amino acid side groups on their surfaces. These fixed arrays can selectively bind to complementary tissue surfaces, thus explaining the observed mutant-dependent tissue tropisms and variability of TTR amyloid disease phenotypes [17–21]. Fibrils with a random sequence of wt and v molecules can cause TTR amyloidosis by non-selective binding, but disease symptoms from random sequence fibrils are expected to occur very late in life and show little or no selectivity of affected tissues compared with fixed-sequence fibrils.

Structured proto-fibrils with a mix of wt and v molecules occur if binding of the molecules is selective, i.e., if the interactions differ between wt:wt, wt:v and v:v. Cryo-EM studies provide indirect evidence for this by showing that interactions between amino acid side groups structure amyloids. Steinebrei et al. [22] found, “The stack of protein layers is mainly stabilized by backbone-backbone hydrogen bonds of the beta sheets. Additionally, there are stabilizing interactions between side chains. Lys15 builds a salt bridge with the Asp18. Glu63 builds a salt bridge with the Lys35 of the n + 1 Layer, which would stabilize the first region with the second. Thr123 builds a salt bridge with Arg104 which would stabilize the loop at the C-terminal end. Two distinct additional density spots separated from the core peptide density can be found. One of these spots could be explained by an alternative histidine side chain conformation in the N-terminal region. The other spot between the N-terminal arch and the C-terminal segment is not assignable.”

The hypothesized requirement for the existence of a complementary tissue site for expression of a particular ATTR amyloid disease phenotype to occur is supported by the relatively low penetrance of ATTR amyloidosis. Although the TTR protein is present in everybody’s blood, ATTR amyloidosis only affects approximately 25% of males over the age of 80 [16, 22] and women are less susceptible. This means that more than 75% of adults either don’t get TTR amyloidosis or they die of something else before symptoms of ATTR amyloidosis become evident. The requirement for fibril-tissue complementarity is also evidenced by the observed differing tissue tropisms and thence disease phenotypes caused by fibrils containing only wildtype or the same TTR variant [23].

The variable age of onset, variable penetrance, and variable tissue tropisms of fibrils that are characteristic of ATTR amyloidosis are evidence of a variable degree of complementarity among people who do get ATTR amyloidosis. If tissue complementarity is high, ATTR amyloidosis is expected to occur relatively early in life and be highly tissue selective. If complementarity is low, ATTR amyloidosis is expected to only occur late in life and will not be particularly tissue selective, but if ATTR amyloidosis does occur, it is expected to produce a progression of disease phenotypes over time.

The model of complementary predicts geographic hotspots of ATTR amyloidosis to occur in groups where complementarity is high. Previous work has assumed geographic clustering of ATTR amyloidosis was due solely to inheritance of a particular mutation in a TTR gene [24–27]. However, this work postulates that expression of the disease phenotype associated with a particular variant also requires a receptive tissue surface. And since both the mutation and tissue surfaces are inherited, both the variant and variable tissue receptor sites must be considered. Assuming tissue surfaces vary among geographic groups and that local marriages make inheritance of tissue surfaces common within a group, the complementarity model predicts that geographic hotspots with high occurrence of TTR amyloidosis and similarity of disease phenotypes will occur [21].

To summarize, because every individual has uniquely structured tissue surfaces that are unique to each tissue [10, pp 127–128], the strength of binding of sequence-structured fibrils to tissues will be a continuum from none to very strong depending on the complementarity between the fibril surface structure and tissue surface structures in the individual. The probability of occurrence of TTR amyloidosis during an individual’s lifetime thus depends on the probability for fibrils with a particular structure, the presence of a tissue surface that is complementary to the fibril surface, and the degree of complementarity [18].

## Fibril complementarity

In the models developed here, fragments of TTR molecules and other β-sheet proteins that may be incorporated into ATTR fibrils can simply be treated as another variant [17]. Also note that the mechanism for structuring fibril surfaces, i.e., fixing the sequence of wt and v, is independent of whether the fibril consists of a linear aggregate, a two-dimensional sheet aggregate, or fragments of β-sheet TTR molecules [2, 15, 22].

Considering the model where proto-fibrils are formed in the blood, circulate, and deposit in complementary tissue sites, blood borne proto-fibrils must be small enough to circulate in the bloodstream, but must be large enough for the surface array of amino acid side groups to recognize binding sites in specific tissues. TTR fibrils with about 10 to 50 protein molecules meet both requirements. Considering the model where fibrils are formed by deposition of single TTR molecules on the tissue surface, molecules are selected by two requirements, complementarity with the molecule on the end of the existing fibril seed, and complementarity with the tissue binding site [26].

The statistical argument developed in the next section gives the probability of forming a perfect or near-perfect sequence of 12 alternating wt and v molecules as a function of the strength of wt:wt, wt:v, and v:v interactions, i.e., formation of structured blood borne proto-fibrils, but the same model also applies to fibrils structured by wt:wt, wt:v, v:v, wt:tissue, and v:tissue interactions. Because variant molecules tend to be removed before secretion into the bloodstream, which increases the ratio of wt to v in serum [9], the statistics of fibril sequencing are also examined as a function of the ratio of wt to v. The probabilities are also limited to alternating wt and v, although we recognize other ordered sequences such as …wt:wt:v:wt:wt:v… are possible. The size of fibrils considered here is limited to 12 molecules because of the rapid increase in computational time required for larger fibrils, but the model is readily expandable to larger fibrils.

The statistical model developed in the following section applies to formation of proto-fibrils in the blood and to fibrils *in situ* on a tissue. Note that selection for structured fibrils that are complementary to the tissue will be stronger in the case of *in situ* formation because placement of a TTR protein molecule in the fibril sequence is selected by both the adjacent molecule and the tissue site.

## Statistical model for formation of structured fibrils

The postulate in this section is that the sequence or pattern of the protein molecules in a fibril is the defining characteristic of surface structure in fibrils. The following illustrates a simple paradigm that tests this postulate by calculating the probability that structured fibrils are the natural outcome of stacking TTR molecules whether that happens in an isolated proto-fibril or on a tissue surface. The illustrations assume a fibril is made of 12 molecules but is easily extended to larger stacks.

Consider two types of molecules, labeled A and B (i.e. wildtype and variant) present in some fixed ratio. To develop the model, we break up the stacking into the following steps:

1. The stack is initiated when two molecules bind. The probability that the molecule of type A is selected to initiate the stack is denoted p. The probability the molecule selected is of type B is simply (1 – p). The probability p is obviously proportional to the A:B ratio. The probability the subsequent molecules selected to be attached to the stack are of type A or type B is also p and (1-p), respectively.
2. There may be a difference in strength of bonding for molecules of type A relative to molecules of type B. Note the strength of bonding may include bonding to a tissue site. The effect of strength of bonding A to A or B to B has the same effect as increasing or decreasing the effective ratio of type A to type B in the serum. Bonding strength differences are included in the modeling by adjusting the parameter p.
3. In addition to the effect of bonding strength, the hypothesis here is that the terminal molecule in the stack or the terminal molecule in the stack and the tissue site, also affects which molecule, A or B, binds next. The probability that the next molecule will bind to the terminal molecule in the stack is modeled as *c* exp(γ) if the two molecules are the same and *c* exp(-γ) if the pair are different. If γ = 0 there is no preference in binding. If the parameter γ is positive then there is an increase in the likelihood of binding for molecules of the same type, e.g. A to A or B to B, and a decrease in the likelihood of binding for molecules of different types, e.g., A to B or B to A. If the parameter γ is negative, the proportional increases and decreases are reversed. The parameter γ allows for flexibility in modeling the effects of the terminal molecules and tissue site on binding probabilities.
4. Given a partial stack of molecules, another molecule is selected and attached to the end of the stack. The probability that the molecule selected is A is also p.
5. Except for the terminal molecules, the probability of selection is assumed to be independent of the profile of the stack, i.e. the types of molecules already in the stack have no effect on whether a molecule of a particular type is selected. The binding of molecules in the stack thus only depends on the molecule to which it is to bind or on the molecule and tissue site it is to bind, parameterized by the value of γ.
6. Steps 4 and 5 are repeated until the fibril contains 12 molecules.

The type of molecule in the *m*^*th*^ position from the beginning of the stack is denoted as *σ*_*m*_, where *σ*_*m*_ takes a value of “0" if the molecule is of type A and a “1" if the molecule is of type B. Now, since the order of the molecules matters in determination of the structure of the fibril, the probability model is specific to the sequence of *σ*_*m*_ values. We therefore model the probability of a specific sequence or pattern, (*σ*_*1*_, *σ*_*2*_, …, *σ*_*12*_), as

$$Prob\left(\sigma_{1},\;\;\ldots \;\;,\;\sigma_{12}\right)=c^{*}p^{1-\sigma_{1}}{\left(1-p\right)}^{\sigma_{1}}\Pi_{i=2}^{12}p^{1-\sigma_{i}}{\left(1-p\right)}^{\sigma_{i}}\;\text{exp}\left(\gamma \left(1-2\sigma_{\text{i}}\right)\left(1-2\sigma_{\text{i}-1}\right)\right)$$ (1)

Note that the first two terms on the right side of Eq (1) resemble the Bernoulli probability model used to model outcomes similar to tossing a coin. But, in this case, a heads/tails binary outcome is replaced by the selection of either an A or a B molecule for the first molecule in the fibril. The first two terms after the product sign are also Bernoulli probabilities for selection of molecules after the first. The multiplication of these terms for each selection is because, unlike the tossing of a coin paradigm, the order in which the outcomes of selecting an A or a B is important. The parameter c is the proportionality constant so the sum of the right-hand side of Eq (4) over all possible sequences of σ values totals 1. When a fibril is complete, there is a specific, distinguishable sequence of type A and type B molecules that comprises the fibril structure.

To look for the sequence or structure in the fibril, the probability for each sequence is plotted. Arranged in order, the sequence (*σ*_*1*_, *σ*_*2*_, …, *σ*_*12*_) can be viewed as a binary representation of a unique integer since the *σ*_*m*_ take on values of either 0 or 1. The unique number, *M*_*σ*_, is given as

$$M_{\sigma }=\sum_{i=0}^{11}(\sigma_{i+1})2^{i}$$ (2)

For example, a sequence of all molecules of type B corresponds to all *σ*_*i*_ values being zero. This is a binary representation of the number 0. Similarly, a sequence of all molecules of type A is a binary sequence of all “1” and represents the integer 4096. Any sequence of *σ*_*i*_, values different than these two extremes will represent an integer, using Eq (2), between these two extremes. The plot of the likelihood or expected frequency of each of the integers is plotted together with a horizontal line assuming that p = 0.5 and γ = 0. This line represents the expected frequency of each of the patterns when each pattern (and each integer) is equally likely, i.e., there is no selectivity.

**Fig 1:**
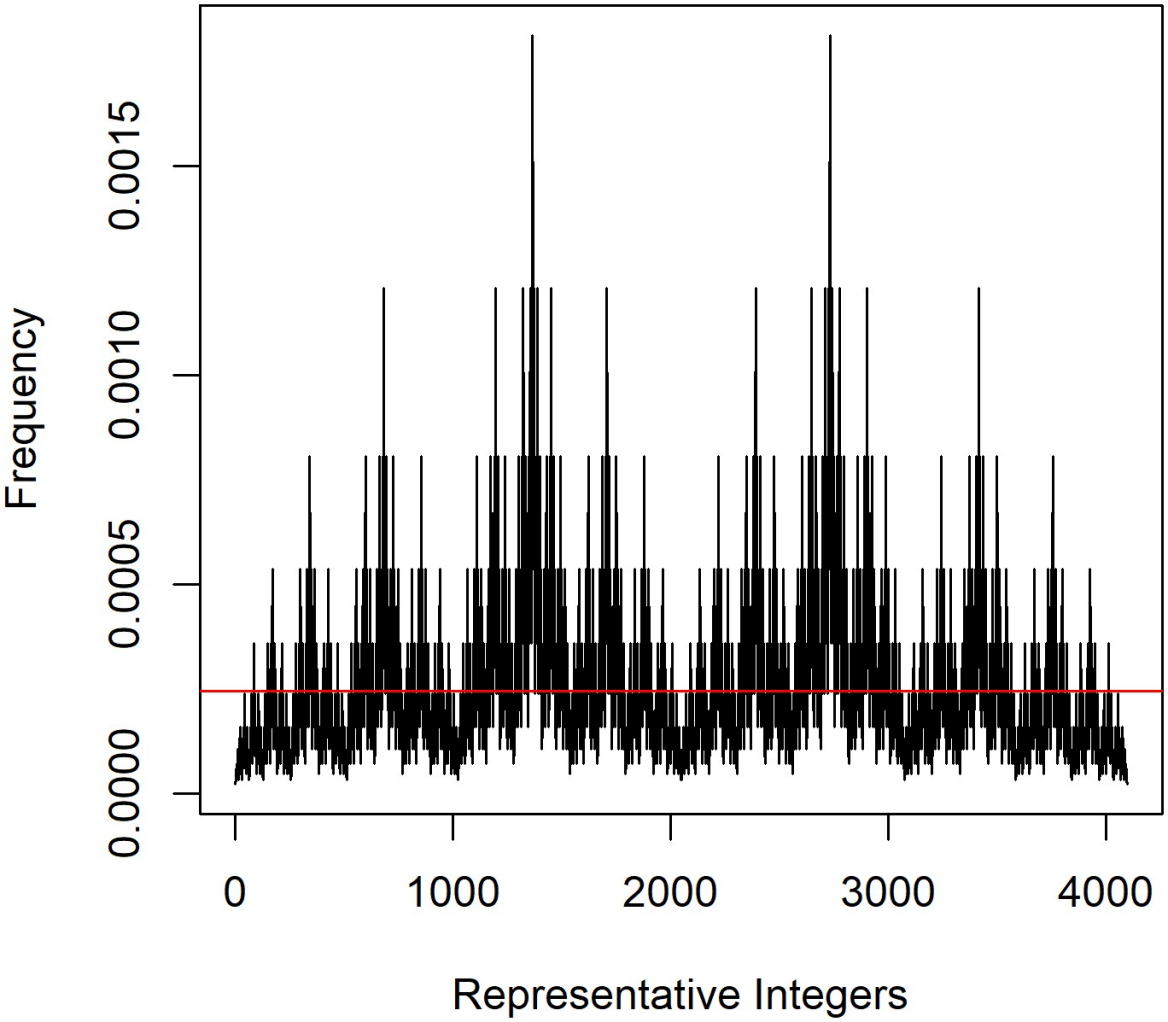
Frequency of perfectly alternating fibrils assuming an odds ratio of 1.5 and equal concentrations of wt and v molecules.

Now, suppose the amino acid side chain in the variant, B, makes it more favorable to attach to a wildtype molecule, A. Thus, if the additional molecule to be attached to a growing fibril is a B, and the end molecule is an A, then the probability of binding will be increased, because the pair of molecules “favor” this binding. Similarly, if a type B molecule is selected, the probability that it will attach to a type A at the end of the fibril is also increased over what it would have been absent the mutation. In comparison to the binding probability, this results in the likelihood of binding between identical molecules, A to A or B to B, to decrease. This is modeled by making γ<0. For example, if γ = -0.2027 in Eq (2), the likelihood of an A to B attachment (given molecules A and B are selected and aligned) is 50% higher than A to A or B to B (exp(0.2027) = 1.5*exp(-0.2027)). This represents odds of 1.5 to 1 of binding or an odds ratio of 1.5. With an assumed 50% increase in the likelihood of successful binding of an A:B pair, the plot of the proportion of each of the 4096 different sequences is given in Fig 1. The red line represents the frequency under random binding and an equal mix of A and B. The two major spikes in Fig 1 correspond to the two integers formed by the binary sequences (1, 0, 1, 0, 1, 0, 1, 0, 1, 0, 1, 0) and (0, 1, 0, 1, 0, 1, 0, 1, 0, 1, 0, 1), respectively. These patterns represent the two dominant fibrils with 12 molecules that are perfectly structured as alternating between type A and type B. The second set of spikes, at about two thirds the height of the two tallest, are the 24 different sequences formed by replacing the position of one type A with that of one type B or vice versa. Comparing the heights of the two spikes in Fig 1 with the reference state, the likelihood of a perfect sequence with a 50% increase in the binding probability for A to B has increased the likelihood of a perfectly alternating fibril from the baseline rate of 2 chances in 4096 or 0.00049 for each of the two different orders to approximately 0.0018 for each. This represents an overall increase of approximately 7.37 times the reference state rate.

**Fig 2:**
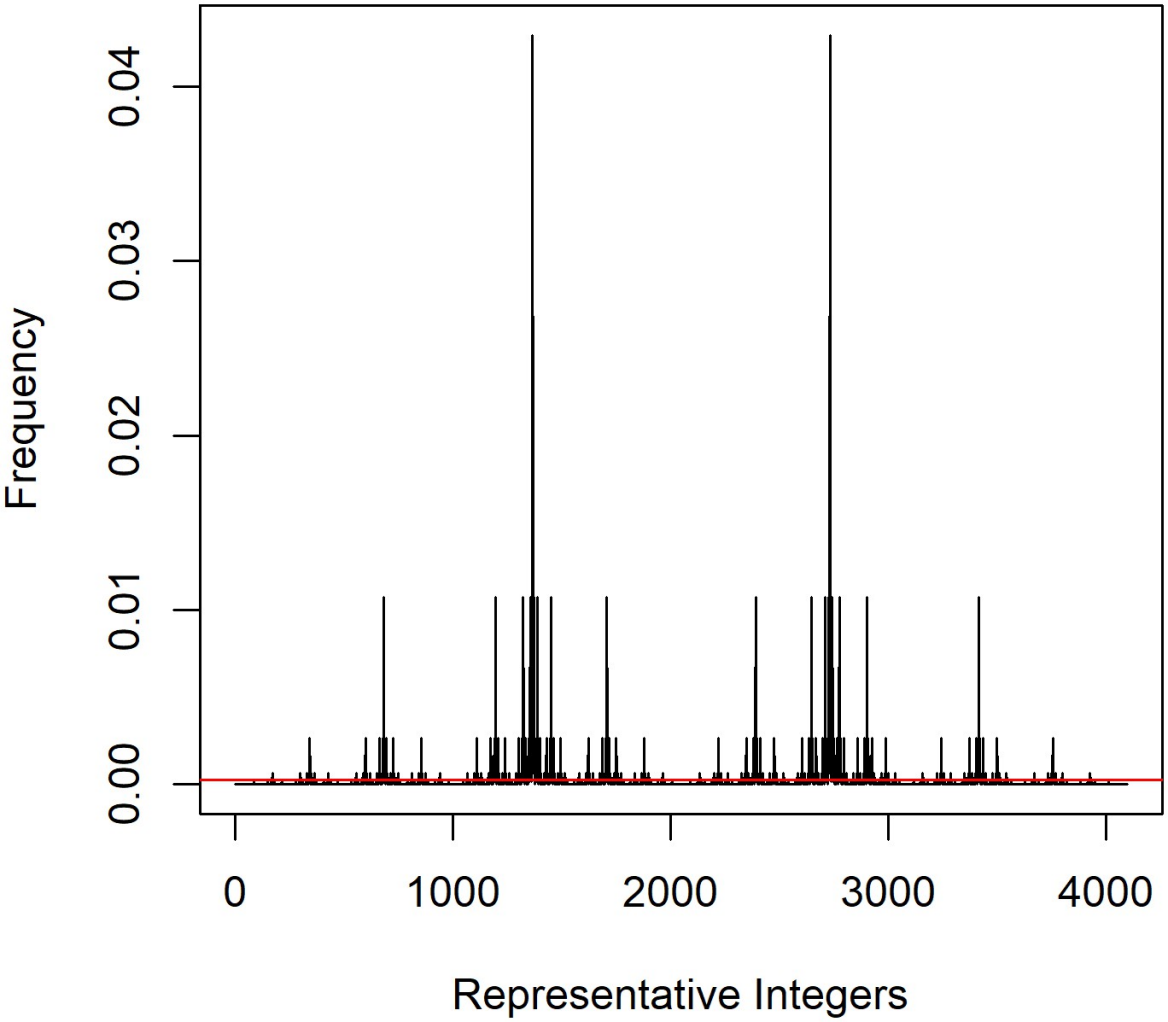
Frequency of perfectly alternating fibrils assuming an odds ratio of 4 and equal concentrations of wt and v molecules.

Fig 2 shows the relative frequency of the representative integers if the binding probability of A to B is increased by a factor of 4. The general pattern is like that of Fig 1 but with more extreme frequencies. In this case, the increase in the likelihood of a perfect alternating fibril has increased from 2 chances in 4096 (0.00049, overall) to 0.086, or over 175 times the reference state of a random sequence.

The above comparisons show the increase in perfectly alternating fibrils relative to the reference state rate. From the blue line in Fig 1 we can determine how many of all fibrils will be perfectly alternating. For example, with a 1:4 ratio (an odds ratio of 4), approximately 10% of all fibrils made will be perfectly alternating. If the mutation increases the binding probability for the pair A:B by a factor of 10, over 30% of all fibrils made will be perfectly alternating.

## Results of statistical modeling

**Fig 3:**
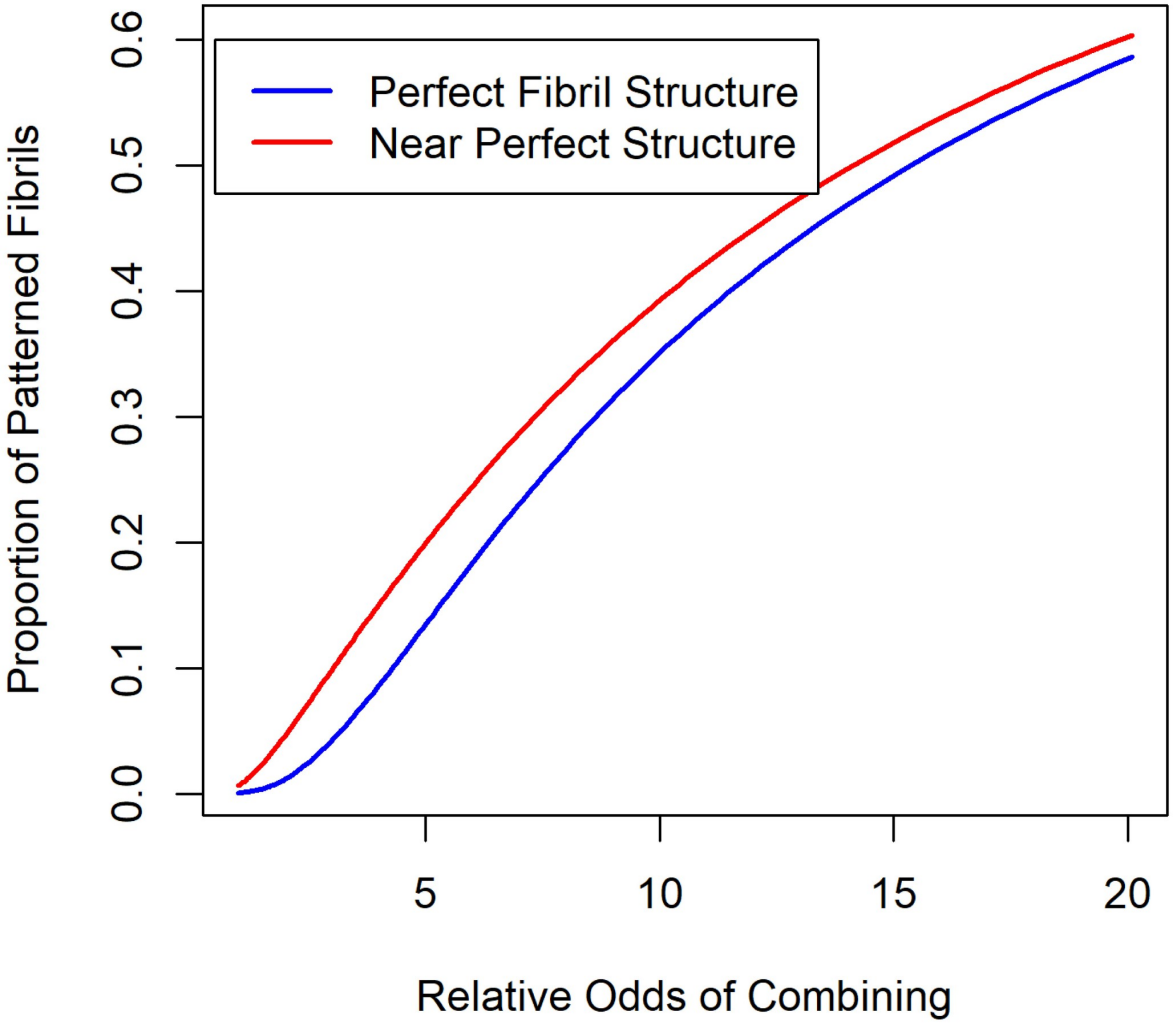
The fraction of the 4096 possible combinations of 12 molecules that have a perfect repeating pattern of alternating wt and v molecules (blue) or have one error (red) as a function of the odds of an added molecule being the correct one. The ratio wt:v is assumed to be 1:1.

Fig 3 gives the results of these calculations when the ratio of wt to v is 1:1. The proportion of fibrils with a perfect or near perfect alternating sequence of wt and v molecules rises surprisingly fast with increasing odds of binding the “correct” molecule. The “odds” are defined as the ratio of the probability of getting the “correct” molecule to make a perfect sequence to the probability of getting the “wrong” molecule in the sequence. Fig 3 thus shows a mutation that improves the fit (or increases the non-covalent bond energy) can easily produce structured fibrils in sufficient concentration to cause tissue-specific amyloidosis.

**Fig 4:**
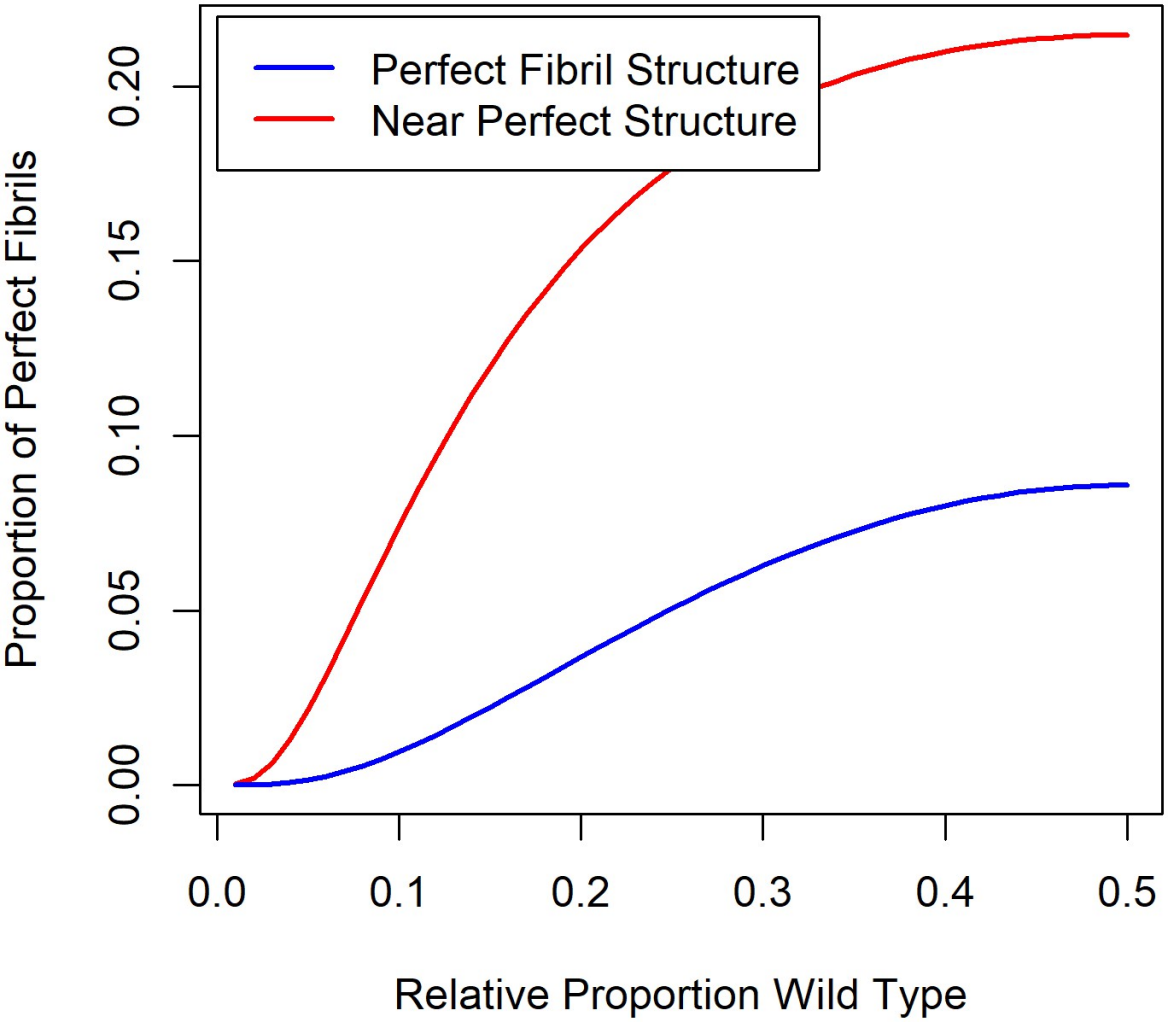
The fraction of the 4096 possible combinations of 12 molecules that have a perfect repeating pattern of alternating wt and v molecules (blue) or have one error (red) as a function of the ratio v:wt. The odds of adding the correct molecule is set at 4:1.

Fig 4 illustrates the likelihood of an alternating sequence as a function of the ratio of v to wt. With odds of only 4:1 for getting the correct molecule, the proportion of perfect alternating fibrils only reaches a maximum of about 5%. However, the proportion of fibrils with only one non-alternating pair exceeds 15% at v:wt ratios >0.2.

The equivalence of “fit” and “bond energy” for binding TTR molecules together and to tissues is discussed below; a better “fit” is shown to be positively correlated with an increased non-covalent “bond energy”. To equate the results of the statistical analysis to a “bond energy”, the “odds” can be related to an energy scale by assuming the “odds” are equivalent to the equilibrium constant, K_eq_, for the reaction to replace a wt molecule on the end of a fibril with a v molecule.


$$\text{Fibril-wt}+\text{v}\left(\text{aq}\right)=\text{Fibril-v}+\text{wt}\left(\text{aq}\right)$$ (3)

Assuming Fibril-v and Fibril-wt are insoluble phases suspended in the solution or bound to a tissue, their activities = 1, and the equilibrium constant for Eq 3 can be written as

$${\text{K}}_{\text{eq}}=\left[\text{wt}\left(\text{aq}\right)\right]/\left[\text{v}\left(\text{aq}\right)\right]=\text{odds}\;\text{ratio}$$ (4)

**Fig 5:**
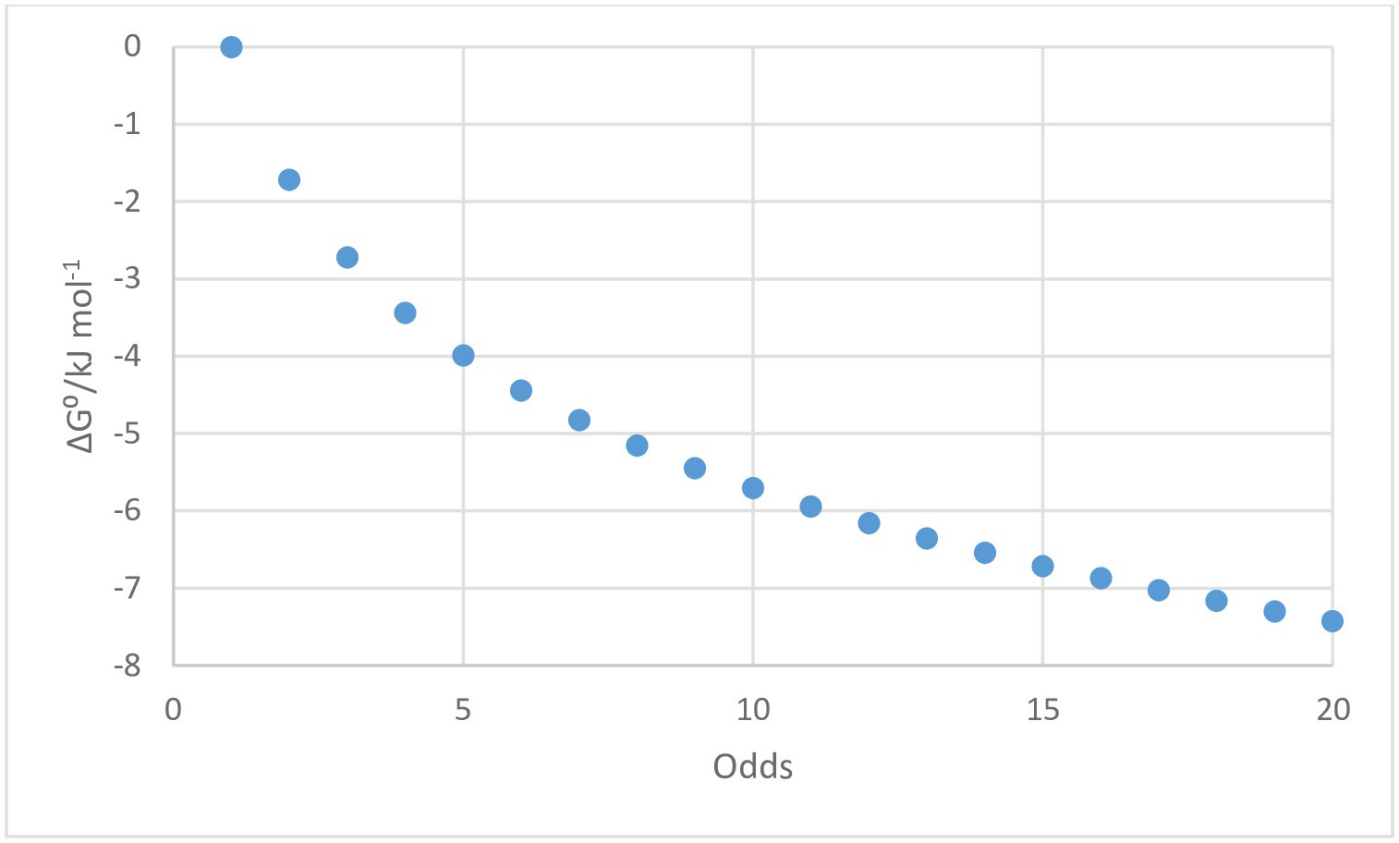
Interaction energies between wt and v molecules as a function of the “odds ratio” assumed in the statistical analysis of structured fibril formation.

The square brackets in the equation indicate the equilibrium concentrations of the molecules when a solution initially composed of equal concentrations of wt(aq) and v(aq) is allowed to go to equilibrium with an equivalent amount of fibrils with one less molecule. K_eq_ is related to the standard Gibbs energy change, ΔG°, by

$$\Delta \text{G}{}^{\circ}={\text{-RTlnK}}_{\text{eq}}=\text{-RTln}”\text{odds}”$$ (5)

which provides the relation shown in Fig 5 between bond energy, as ΔG°, and the odds ratio. Note that an “odds” ratio of 2:1 is approximately equivalent to the Gibbs energy for formation of one—OH∙∙∙O- hydrogen bond in water, i.e. 2 kJ mol^-1^ [28]. This is not to suggest that hydrogen bonding is necessarily the cause of differences in bond strengths between wt-wt, wt-v, and v-v β-sheet TTR molecules or between β-sheet TTR molecules and tissues, but serves as a familiar reference point for the effects of the sum of changes in non-covalent bonding due to changes in the ‘fit’ or complementarity between the molecules. It should also be apparent that a mutation that increases wt-v bond strength relative to wt-wt and v-v bonds will increase the likelihood of structured fibrils and that a mutation that increases the relative strength of v-v bonds will decrease the likelihood of structured fibrils with an alternating sequence of wt and v molecules.

The results of the statistical analysis thus show that a fixed sequence of wt and v protein molecules in fibrils is highly probable as a natural outcome of stacking wt and v β-sheets and that the assumed probabilities are in a very feasible range of interaction energies. The statistical model shows that changes in the “fit” between wt and v molecules, as represented by the odds in the calculations, can significantly increase or decrease the probability of structured fibrils. It is also apparent that structured fibrils of various lengths and sequences other than alternating wt and v, e.g. …wt-wt-v-wt-wt-v-wt-wt…, etc., can also be formed. Because the surface array of amino acid side groups depends on the variant and the sequence, differing tissue tropisms and differing disease phenotypes are possible with differing sequences. Also, because different ordered sequences may co-occur in significant amounts, more than one kind of tissue may be simultaneously affected.

## Discussion and conclusions

The fundamental assumptions in the proposed model for amyloid formation may be stated as follows: For TTR amyloidosis to occur, molecules of cross-β-sheet TTR protein molecules must be strongly bound to each other and to tissue surfaces through multiple weak non-covalent bonds. Since tissue surfaces are well-known to be highly ordered, genetically determined, and unique to each tissue and to every individual, the bonding surface of amyloids must also be highly structured. And since the strength of fibril binding to a tissue surface is a continuum with increasing strength as the complementarity and number of bonds increases, the model predicts high complementarity is required to achieve the strength of bonding required to explain the insolubility of fibrils. The requirement for strong tissue complementarity which causes tissue selectivity explains the variability of disease phenotypes expressed. The relatively low penetrance of TTR amyloid diseases is likely caused by a lack of strong complementarity in most individuals. In those individuals who do contract TTR amyloidosis, the model predicts the age of initiation of symptoms to increase as complementarity decreases.

The concepts of structured fibrils and complementarity (i.e., the number of attractive interactions minus the number of repulsive interactions) between the surface groups on fibrils and on tissues are key developments of this work. Although complementarity can only be inferred in this work, complementarity is a well-known common organizing principle in biology, e.g., complementarity between strands in double-stranded DNA, production of sequenced RNA by complementarity to DNA, sequencing of proteins by m-RNA, and selectivity of enzyme catalysis by complementarity between the binding site and the substrate.

Mutation of a TTR gene necessarily changes the groups on the surface of fibrils and the model developed here shows mutations can result in a continuum of changes in fibril-tissue binding affinities that cause major changes in tissue specificity of fibril binding and ultimately change the age of onset and amyloid disease phenotype [20]. Multiple combinations of v plus wt fibrils in different ratios may be assembled in fibrils, and each of these combinations has the potential to bind with different complementary tissues. Therefore, the presence of TTRv has the potential to produce fibrils with affinities for multiple tissue sites that would not bind TTRwt. A random sequence of wt and v molecules in an amyloid fibril may cause TTR amyloidosis by non-selective binding, but disease symptoms are expected to occur very late in life and not be limited to a particular tissue or tissues.

The model developed here predicts structured amyloid fibrils present mutant-dependent arrangements of amino acid side groups on their surfaces that selectively bind to different tissues, which together with tissue surface variability among individuals, explains the observed mutant-dependent tissue tropisms and consequent TTR amyloid disease phenotypes. Variation in the degree of complementarity explains the large variation observed in age of onset and disease progression.

This paper focuses on TTR amyloidosis, but several other blood-borne proteins also cause amyloid diseases and the principles developed in this paper are generally applicable. The physical properties of the 30 or so amyloidogenic human proteins or protein segments favor spontaneous refolding and aggregation into cross-β structures which are a common feature of all amyloid diseases. Some precursor proteins form homogenous amyloids and others that readily accept mutation or modification form heterogeneous amyloids. This paper thus lays a foundation for understanding the effects of fibril structure and consequent disease phenotypes for all human amyloid diseases. Thus, the real significance of this work lies in the understanding of the process that forms fibrils and binds fibrils to tissues which may lead to new possibilities for interrupting the process and preventing or curing ATTR amyloidosis and other human amyloid diseases.

## Abbreviations

TTR: transthyretin
v: variant
wt: wildtype
